# Supplementary material for: Computationally-driven identification of antibody epitopes
Source: eLife. 2017 Dec 4;6:e29023. doi: 10.7554/eLife.29023 (PMC5739537; doi:10.7554/eLife.29023)
Supplement: Supplementary file 3. [file elife-29023-supp3.pdf]

TZ47 scFv
QVQLQQSGAELMKPGASVKLSCKATGYTFTGYWIEWIKQRPGHGLEWIGEILPGTGSTNYNEKFKGKATFTADTSS
NTAYMQLSSLTTEDSAIYYCAIPGPMDYWGQGTSVTVSSAGGGGSGGGGSGGGGSEFDIKMTQSPSSMYASLGER
VTITCKASQDINSYLSWFQQKPGKSPKTLIYRANRLVDGVPSRFSGSGSGQDYSLTISSLEYEDMGIYYCLQYDEFPYTF
GGGTKLEIK

TZ47-specific Designs
Summary of TZ47-specific Design Mutations

Design	Mutations
TZ47-Ag1	F24Y, N26Q, W75E
TZ47-Ag2	F161D, I165Q, V202T
TZ47-Ag3	T48K, K51E, V53H
TZ47-Ag4	M131E, N134G, S194H

*Mutations annotated in red

TZ47-Ag1
GDLKVEMMAGGTQITPLNDNVTIYCQIFYSQPLNITSMGITWFWKSLTFDKEVKVFEFFGDHQEAFRPGAIVSPERL
KSGDASLRLPGIQLEEAGEYRCEVVVTPLKAQGTVQLEVVASPASRLLLDQVGMKENEDKYMCESSGFYPEAINITW
EKQTQKFPHPIEISEDVITGPTIKNMDGTFNVTSCLKLNSSQEDPGTVYQCVVRHASLHTPLRSNFTLTAARHSLSETE
KTDNFS

TZ47-Ag2
GDLKVEMMAGGTQITPLNDNVTIFCNIFYSQPLNITSMGITWFWKSLTFDKEVKVFEFFGDHQEAFRPGAIVSPWR
LKSGDASLRLPGIQLEEAGEYRCEVVVTPLKAQGTVQLEVVASPASRLLLDQVGMKENEDKYMCESSGFYPEAINIT
WEKQTQKDPHPQEISEDVITGPTIKNMDGTFNVTSCLKLNSSQEDPGTTYQCVVRHASLHTPLRSNFTLTAARHSLS
ETEKTDNFS

TZ47-Ag3
GDLKVEMMAGGTQITPLNDNVTIFCNIFYSQPLNITSMGITWFWKSLKFDEEHKVFEFFGDHQEAFRPGAIVSPWR
LKSGDASLRLPGIQLEEAGEYRCEVVVTPLKAQGTVQLEVVASPASRLLLDQVGMKENEDKYMCESSGFYPEAINIT
WEKQTQKFPHPIEISEDVITGPTIKNMDGTFNVTSCLKLNSSQEDPGTVYQCVVRHASLHTPLRSNFTLTAARHSLSE
TEKTDNFS

TZ47-Ag4
GDLKVEMMAGGTQITPLNDNVTIFCNIFYSQPLNITSMGITWFWKSLTFDKEVKVFEFFGDHQEAFRPGAIVSPWR
LKSGDASLRLPGIQLEEAGEYRCEVVVTPLKAQGTVQLEVVASPASRLLLDQVGEKEGEDKYMCESSGFYPEAINITW
EKQTQKFPHPIEISEDVITGPTIKNMDGTFNVTSCLKLNHSQEDPGTVYQCVVRHASLHTPLRSNFTLTAARHSLSETE
KTDNFS

--- --- --- --- --- --- ---

PB11 scFv
QVQLVQSGAEVKKPGASVKISCKASGYTLGTYTLHWMRQAPGQRIEWMGWINPGNGYTKYSQRFQGRVTINRDT
SATTTYMELSSLRSEDTAVYFCATDRAPVRRAFDIWGQGTMVTVSSGILGSGGGGSGGGGSGGGGSQSVLTQPAS
VSGSPGQSVTISCTGTSSDIGSYKFVSWYQHHPGKAPKLMIYDVTQRPSGVPYRFSGSKSGNTASLTISGLQAEDEAD
YYCCSYAGDYTYALFGGGTQLTVLS

PB-specific Designs
Summary of PB-specific Design Mutations

**Design**	**Mutations**
PB-Ag1	M7V, Q109V, Q113L
PB-Ag2	F28H, Y29D, R76G
PB-Ag3	A65T, F66T, G88R
PB-Ag4	T153K, V171I, R208E
PB-Ag5	N193K, S194A, Q196V

*Mutations annotated in red

PB-Ag1

GDLKVEVMAGGTQITPLNDNVTIFCNIFYSQPLNITSMGITWFWKSLTFDKEVKVFEFFGDHQEAFRPGAIVSPWRL
KSGDASLRLPGIQLEEAGEYRCEVVVTPLKAVGTVLLEVVASPASRLLLDQVGMKENEDKYMCESSGFYPEAINITWE
KQTQKFPHPIEISEDVITGPTIKNMDGTFNVTSCLKLNSSQEDPGTVYQCVVRHASLHTPLRSNFTLTAARHSLSETEK
TDNFS

PB-Ag2

GDLKVEMMAGGTQITPLNDNVTIFCNIHDSQPLNITSMGITWFWKSLTFDKEVKVFEFFGDHQEAFRPGAIVSPWG
LKSGDASLRLPGIQLEEAGEYRCEVVVTPLKAQGTVQLEVVASPASRLLLDQVGMKENEDKYMCESSGFYPEAINIT
WEKQTQKFPHPIEISEDVITGPTIKNMDGTFNVTSCLKLNSSQEDPGTVYQCVVRHASLHTPLRSNFTLTAARHSLSE
TEKTDNFS

PB-Ag3

GDLKVEMMAGGTQITPLNDNVTIFCNIFYSQPLNITSMGITWFWKSLTFDKEVKVFEFFGDHQETTRPGAIVSPWRL
KSGDASLRLPRIQLEEAGEYRCEVVVTPLKAQGTVQLEVVASPASRLLLDQVGMKENEDKYMCESSGFYPEAINITW
EKQTQKFPHPIEISEDVITGPTIKNMDGTFNVTSCLKLNSSQEDPGTVYQCVVRHASLHTPLRSNFTLTAARHSLSETE
KTDNFS

PB-Ag4

GDLKVEMMAGGTQITPLNDNVTIFCNIFYSQPLNITSMGITWFWKSLTFDKEVKVFEFFGDHQEAFRPGAIVSPWR
LKSGDASLRLPGIQLEEAGEYRCEVVVTPLKAQGTVQLEVVASPASRLLLDQVGMKENEDKYMCESSGFYPEAINIK
WEKQTQKFPHPIEISEDIITGPTIKNMDGTFNVTSCLKLNSSQEDPGTVYQCVVEHASLHTPLRSNFTLTAARHSLSET
EKTDNFS

PB-Ag5

GDLKVEMMAGGTQITPLNDNVTIFCNIFYSQPLNITSMGITWFWKSLTFDKEVKVFEFFGDHQEAFRPGAIVSPWR
LKSGDASLRLPGIQLEEAGEYRCEVVVTPLKAQGTVQLEVVASPASRLLLDQVGMKENEDKYMCESSGFYPEAINIT
WEKQTQKFPHPIEISEDVITGPTIKNMDGTFNVTSCLKLKASVEDPGTVYQCVVRHASLHTPLRSNFTLTAARHSLSET
EKTDNFS

<u>Multi-Ab Designs</u>

Summary of MULTI-specific Design Mutations

Design	Mutations
MULTI-1	N34D, D61N, W75E
MULTI-2	F43Y, T48K, F49D
MULTI-3	V55L, F66T, G88R
MULTI-4	M131E, N134E, N193K
MULTI-5	A149H, R208E, A210E
MULTI-6	T153K, R208E, H213S

*Mutations annotated in red

MULTI-1

GDLKVEMMAGGTQITPLNDNVTIFCNIFYSQPLDITSMGITWFWKSLTFDKEVKVFEFFGNHQEAFRPGAIVSPERL
KSGDASLRLPGIQLEEAGEYRCEVVVTPLKAQGTVQLEVVASPASRLLLDQVGMKENEDKYMCESSGFYPEAINITW
EKQTQKFPHPIEISEDVITGPTIKNMDGTFNVTSCLKLNSSQEDPGTVYQCVVRHASLHTPLRSNFTLTAARHSLSETE
KTDNFS

MULTI-2

GDLKVEMMAGGTQITPLNDNVTIFCNIFYSQPLNITSMGITWYWKSLKDDKEVKVFEFFGDHQEAFRPGAIVSPWR
LKSGDASLRLPGIQLEEAGEYRCEVVVTPLKAQGTVQLEVVASPASRLLLDQVGMKENEDKYMCESSGFYPEAINIT
WEKQTQKFPHPIEISEDVITGPTIKNMDGTFNVTSCLKLNSSQEDPGTVYQCVVRHASLHTPLRSNFTLTAARHSLSE
TEKTDNFS

MULTI-3

GDLKVEMMAGGTQITPLNDNVTIFCNIFYSQPLNITSMGITWFWKSLTFDKEVKLFEFFGDHQEATRPGAIVSPWRL
KSGDASLRLPRIQLEEAGEYRCEVVVTPLKAQGTVQLEVVASPASRLLLDQVGMKENEDKYMCESSGFYPEAINITW
EKQTQKFPHPIEISEDVITGPTIKNMDGTFNVTSCLKLNSSQEDPGTVYQCVVRHASLHTPLRSNFTLTAARHSLSETE
KTDNFS

MULTI-4
GDLKVEMMAGGTQITPLNDNVTIFCNIFYSQPLNITSMGITWFWKSLTFDKEVKVFEFFGDHQEAFRPGAIVSPWR
LKSGDASLRLPGIQLEEAGEYRCEVVVTPLKAQGTVQLEVVASPASRLLLDQVGEKEEEDKYMCESSGFYPEAINITW
EKQTQKFPHPIEISEDVITGPTIKNMDGTFNVTSCLKLKSSQEDPGTVYQCVVRHASLHTPLRSNFTLTAARHSLSETE
KTDNFS

MULTI-5
GDLKVEMMAGGTQITPLNDNVTIFCNIFYSQPLNITSMGITWFWKSLTFDKEVKVFEFFGDHQEAFRPGAIVSPWR
LKSGDASLRLPGIQLEEAGEYRCEVVVTPLKAQGTVQLEVVASPASRLLLDQVGMKENEDKYMCESSGFYPEHINIT
WEKQTQKFPHPIEISEDVITGPTIKNMDGTFNVTSCLKLNSSQEDPGTVYQCVVEHESLHTPLRSNFTLTAARHSLSET
EKTDNFS

MULTI-6
GDLKVEMMAGGTQITPLNDNVTIFCNIFYSQPLNITSMGITWFWKSLTFDKEVKVFEFFGDHQEAFRPGAIVSPWR
LKSGDASLRLPGIQLEEAGEYRCEVVVTPLKAQGTVQLEVVASPASRLLLDQVGMKENEDKYMCESSGFYPEAINIK
WEKQTQKFPHPIEISEDVITGPTIKNMDGTFNVTSCLKLNSSQEDPGTVYQCVVEHASLSTPLRSNFTLTAARHSLSET
EKTDNFS
