# Supplementary material for: Computationally-driven identification of antibody epitopes
Source: eLife. 2017 Dec 4;6:e29023. doi: 10.7554/eLife.29023 (PMC5739537; doi:10.7554/eLife.29023)
Supplement: Transparent reporting form [file elife-29023-transrepform.docx]

***eLife’s* transparent reporting form**

We encourage authors to provide detailed information *within their submission* to facilitate the interpretation and replication of experiments. If you have any questions, please contact us: [editorial@elifesciences.org](mailto:editorial@elifesciences.org).

**Sample-size estimation**

- You should state whether an appropriate sample size was computed when the study was being designed
- You should state the statistical method of sample size computation and any required assumptions
- If no explicit power analysis was used, you should describe how you decided what sample (replicate) size (number) to use

Please outline where this information can be found within the submission (e.g., page numbers or figure legends), or explain why this information doesn’t apply to your submission:

This doesn’t really apply to our work unless referring to mean calculated from flow cytometry binding data: within each well of stained cells, samples were collected until 25,000 total events fell within a Calcein-positive “Live cell” gate or until the sample volume ran out.

**Replicates**

- You should report how often each experiment was performed
- You should include a definition of biological versus technical replication
- The data obtained should be provided and sufficient information should be provided to indicate the number of independent biological and/or technical replicates
- If you encountered any outliers, you should describe how these were handled
- Criteria for exclusion/inclusion of data should be clearly stated
- High-throughput sequence data should be uploaded before submission, with a private link for reviewers provided (these are available from both GEO and ArrayExpress)

Please outline where this information can be found within the submission (e.g., page numbers or figure legends), or explain why this information doesn’t apply to your submission:

Replicate information is included in figure legends for Figs 2 & 3.

**Statistical reporting**

- Statistical analysis methods should be described and justified
- Raw data should be presented in figures whenever informative to do so (typically when N per group is less than 10)
- For each experiment, you should identify the statistical tests used, exact values of N, definitions of center, methods of multiple test correction, and dispersion and precision measures (e.g., mean, median, SD, SEM, confidence intervals; and, for the major substantive results, a measure of effect size (e.g., Pearson's r, Cohen's d)
- Report exact p-values wherever possible alongside the summary statistics and 95% confidence intervals. These should be reported for all key questions and not only when the p-value is less than 0.05.

Please outline where this information can be found within the submission (e.g., page numbers or figure legends), or explain why this information doesn’t apply to your submission:

Raw data files are all described below in the “Additional data files” section.

(For large datasets, or papers with a very large number of statistical tests, you may upload a single table file with tests, Ns, etc., with reference to page numbers in the manuscript.)

**Additional data files (“source data”)**

- We encourage you to upload relevant additional data files, such as numerical data that are represented as a graph in a figure, or as a summary table
- Where provided, these should be in the most useful format, and they can be uploaded as “Source data” files linked to a main figure or table
- Include model definition files including the full list of parameters used
- Include code used for data analysis (e.g., R, MatLab)
- Avoid stating that data files are “available upon request”

Please indicate the figures or tables for which source data files have been provided:

- All the designs presented in this work were generated using the EpiSweep software package (<http://www.cs.dartmouth.edu/~cbk/episweep/register.php>) with default “DisruPPI” design strategy.
- The scoring matrix for binding disruption used in this study (INT5) was obtained from Table SI-1 in “Pons et al. (2011) JCIM, 51 (2) 370-377”.
- ClusPro server was used to generate docking models with “Antibody Mode” and “Automatically Mask non-CDR regions” options in Advanced Options.
- PIGS server was used to model antibody structures with “Same Antibody”, “Graft all loops” and “Transfer conserved + SCWRL 4.0”.
- PyFREAD was used to model CDR loops with default cut-off setting (the environment-specific substitution score >= 25).
- Tinker minimization parameter file is provided in supplementary code file 1.
- OSPREY parameter files are provided in supplementary code file 2 as a compressed zip file with an example.
- Source data for Figure 2 is provided in fig2-source-data.xlsx.
- Source data for Figure 3 is provided in fig3-source-data.xlsx
- Raw data for Figure 4 and Figure 4-Figure Supplement 1 are associated with Table 4.
- Raw data for “Figure 4-Figure Supplement 2” is provided as a compressed zip file (fig4_fig_sup2-source-data.zip).
- Raw data for “Figure 4-Figure supplement 3” is provided as a CSV file (fig4_fig_sup3-source-data.csv).
- Raw data for “Figure 4-Figure Supplement 4” is provided as a compressed zip file (fig4_fig_sup4-source-data.zip).
- Raw data for “Figure 5A, B and C” is provided as a compressed zip file (fig5-source-data.zip).
- Figure 5A was reproduced from RGB values of Figure 2 in “Sela-Culang et al. (2014) Structure, 22 (4) 646-657”.
- Figure 5 CDR information was obtained from Table S2 in “Sela-Culang et al. (2014) Structure, 22 (4) 646-657”.
- Raw data for “Figure 5-Figure supplement 1” is provided as a CSV file (fig5_fig_sup1-source-data.csv).
- Raw data for “Figure5-Figure supplement 3” is provided as a compressed zip file (fig5_fig_sup3-source-data.zip).
- Raw data for Figure 6 is provided as a compressed zip file (fig6-source-data.csv).
- Detailed information for Table 4 is provided as a CSV file (table4-source-data.csv).
